# Identification and characterization of extracellular vesicles from red cells infected with *Babesia divergens* and *Babesia microti*

**DOI:** 10.3389/fcimb.2022.962944

**Published:** 2022-10-07

**Authors:** Divya Beri, Marilis Rodriguez, Manpreet Singh, Yunfeng Liu, Giselle Rasquinha, Xiuli An, Karina Yazdanbakhsh, Cheryl A. Lobo

**Affiliations:** ^1^ Department of Blood-Borne Parasites, Lindsley F. Kimball Research Institute, New York Blood Center, New York, NY, United States; ^2^ Department of Complement Biology, Lindsley F. Kimball Research Institute, New York Blood Center, New York, NY, United States; ^3^ Department of Biology, Georgetown University, Washington, DC, United States; ^4^ Department of Membrane Biology, Lindsley F. Kimball Research Institute, New York Blood Center, New York, NY, United States

**Keywords:** *Babesia*, extracellular vesicles (EV), image flow cytometry, miRNA, microarray

## Abstract

Babesiosis is a zoonosis and an important blood-borne human parasitic infection that has gained attention because of its growing infection rate in humans by transfer from animal reservoirs. *Babesia* represents a potential threat to the blood supply because asymptomatic infections in man are common, and blood from such donors can cause severe disease in certain recipients. Extracellular vesicles (EVs) are vesicles released by cells that contain a complex mixture of proteins, lipids, glycans, and genetic information that have been shown to play important roles in disease pathogenesis and susceptibility, as well as cell–cell communication and immune responses. In this article, we report on the identification and characterization of EVs released from red blood cells (RBCs) infected by two major human *Babesia* species—*Babesia divergens* from *in vitro* culture and those from an *in vivo B. microti* mouse infection. Using nanoparticle tracking analysis, we show that there is a range of vesicle sizes from 30 to 1,000 nm, emanating from the *Babesia*-infected RBC. The study of these EVs in the context of hemoparasite infection is complicated by the fact that both the parasite and the host RBC make and release vesicles into the extracellular environment. However, the EV frequency is 2- to 10-fold higher in *Babesia*-infected RBCs than uninfected RBCs, depending on levels of parasitemia. Using parasite-specific markers, we were able to show that ~50%–60% of all EVs contained parasite-specific markers on their surface and thus may represent the specific proportion of EVs released by infected RBCs within the EV population. Western blot analysis on purified EVs from both *in vivo* and *in vitro* infections revealed several parasite proteins that were targets of the host immune response. In addition, microRNA analysis showed that infected RBC EVs have different microRNA signature from uninfected RBC EVs, indicating a potential role as disease biomarkers. Finally, EVs were internalized by other RBCs in culture, implicating a potential role for these vesicles in cellular communication. Overall, our study points to the multiple functional implications of EVs in *Babesia*–host interactions and support the potential that EVs have as agents in disease pathogenesis.

## Introduction

Membrane-bound vesicles containing proteins, nucleic acids, and lipids have been shown to be secreted by a diverse range of eukaryotic and prokaryotic cells. Although they were initially characterized as cell debris, they are now recognized to play an important role in transferring information among cells that are not in direct contact with each other (Wolf, 1967; Gill et al., 2019). Encased within a vesicle, their contents are protected from enzymatic cleavage and fluctuations in both pH and osmolarity encountered in the environment. Extracellular vesicles (EVs) are composed of a heterogeneous group of cell-derived vesicles including exosomes that range in size from 30 to 150 nm (exosomes) and microvesicles (MVs) that span 150–1,000 nm (Babatunde et al., 2020). The contents, size, and membrane composition of EVs are highly heterogeneous and dynamic and depend on the cellular source, state, and environmental conditions (Yanez-Mo et al., 2015).

Functional roles for EVs have been broadly categorized as regulation of gene expression, signal induction, distribution of catalytic activity, and disposal of cellular debris (Shifrin et al., 2013). Their main mechanism of action is serving as vehicles transporting their effector molecular cargo from one cell to another, resulting in functional consequences for the target recipient cells (Schorey et al., 2015). Sometimes, these signals are targeted to cells within a population, such as quorum sensing in bacteria; at other times, EVs from a donor cell modulate other cell types, with a prime example being immune cells as recipients of microbial signals during infection (Shifrin et al., 2013). The mechanisms of EV interaction with target cells are also diverse, ranging from examples of ligand/receptor-mediated binding, phagocytosis, or direct membrane fusion (Szempruch et al., 2016). The cargo content in EVs has been shown to vary with the cell of origin, suggesting a selective loading mechanism (Xie et al., 2022). In the context of infection, EVs have been shown to be secreted by the infectious agent itself or by the host cells (infected or uninfected), potentially influencing the course of the disease (Marti and Johnson, 2016; Martins and Alves, 2020).

In the last decade, there has been many reports on the release of EVs from major human parasitic pathogens including Plasmodium, Leishmania, Giardia, Trypanosoma, Schistosoma, and Fasciola species (Marti and Johnson, 2016; Moyano et al., 2019). These pathogens have a dual-host life cycle that requires quick adaptation to changing environments, and EVs have been shown to form a key strategy that these parasites use to persist in the human host by regulating host immune responses and provide sensing mechanisms within the parasite population (Cipriano and Hajduk, 2018). In this study, we explored and characterized the EV repertoire of Babesia, a related apicomplexan parasite. Babesiosis is a zoonosis, a disease communicable from animals to man and an important blood-borne human parasitic infection (Ord and Lobo, 2015; Vannier et al., 2015; Lobo et al., 2020). Like the others, Babesia parasites present a complex life cycle spanning two hosts—a tick vector and a mammalian host. Of the five species that cause human disease, *B. divergens* and *B. microti* have received the most attention because of their growing infection rate in humans *via* transfer from animal reservoirs, and as asymptomatic infections in man are common, these can be life threatening in certain blood transfusion recipients like hemoglobinopathic individuals (Lobo et al., 2013; Schmidt et al., 2014; Beri et al., 2021).

The growing interest concerning exosomes in infectious diseases, their accessibility in various body fluids, and their capacity to carry a rich protein content highlights the potential use of EVs as new diagnostic and therapeutic tools (Properzi et al., 2013). Apart from protein, EVs have been shown to contain all types of biomolecules, including carbohydrates, lipids, and nucleic acid. Nucleic acid species found in these vesicles include DNA and both non-coding RNAs and messenger RNAs (Nolte-’T Hoen et al., 2012; Kim et al., 2017). Of particular interest to our study is the presence of microRNAs (miRNAs), which could be transferred to and function in recipient cells.

In this article, we identify and characterize purified EVs secreted by *B. divergens* using an *in vitro* culture system and *B. microti* using an *in vivo* mouse model. Our data show that both Babesia species secrete vesicles that have a size and shape consistent with EVs from other parasites. Quantitative analysis of these vesicles revealed a correlation with parasite infection matrices. Importantly, we show that labeled EVs are taken up by other red blood cells (RBCs) in culture. The presence of Babesia-derived components, including protein on the surface and within these EVs, makes them highly immunogenic, as demonstrated by reactivity with infected human and mouse sera. A detailed miRNA analysis also provides evidence of highly up-regulated miRNA species in EVs from infected cells, pointing to a potential role in influencing disease outcome. Our results provide a rationale for a detailed study of the role of these EVs in the pathogenesis of babesiosis, as well as to understand their mode of serving as a mechanism of parasite survival by mediating communication among infected cells, as has been implicated in other parasite systems (Marti and Johnson, 2016; Correa et al., 2020).

## Materials and methods

### B divergens in vitro culture

*B. divergens* (Bd Rouen 1986 strain) were maintained in human RBCs at 5% hematocrit in complete medium (RPMI-1640; supplemented with 50 μg/ml hypoxanthine, 0.24% (v/v) sodium bicarbonate, and 10% human serum) under low oxygen atmosphere (5% O2, 5%CO2, 90% N2) at 37°C, as previously described (Cursino-Santos et al., 2019). A+ RBCs were collected in 10% CPD and washed 3× with RPMI-1640 medium for the complete plasma and white cells removal. Human A+ serum used to prepare culture media were centrifuged at 100,000 × g for 2 h at 4°C to remove exogenous EVs. Parasite proliferation analysis were carried out by flow cytometry. Characterization of parasite morphology and development was performed by Giemsa-stained slides using light microscopy using a 100× objective. Conditioned media or spent media is used to describe the media that is formed by cell growth and remains after culturing of cells, both iRBCs and uRBCs, for 24 h.

### Propagation of *B. microti* in mice

C57BL/6J (000664) were purchased from The Jackson Laboratory (Bar Harbor, ME). Both male and female mice, 9–12 weeks old, were used for this study; animals were housed in microisolator cages in a special pathogen-free facility. The mice were injected with 1 × 108 parasitized cells *via* intraperitoneal route. Once the desired parasitemia was reached, blood from infected BALB/C C57/6J mice (40%–50% parasitemia on day 7/day 8 post invasion) was collected by cardiac puncture in anesthetized mice. For measuring parasitemia, 1 μl blood was withdrawn from the tail. For NanoSight, 20–30 μl of whole blood was drawn by retro-orbital bleeding in anesthetized mice; plasma was obtained and sent for analysis.

All animal studies were approved by the New York Blood Center’s Animal Care and Use Committee.

### Short-term *ex vivo* culture of *B. microti*

Infected blood was collected by retro-orbital bleeding when the parasitemia in the mice was ∼10%. After removing buffy coat, RBC was washed 3× with serum-free RPMI and set into culture at 4% hematocrit for 24 h at 37°C in 1× RPMI supplemented with 367 mM hypoxanthine, 10% fetal bovine serum, and 10 mg/ml gentamycin. Fetal bovine serum used to prepare culture media was centrifuged at 100,000 × g for 2 h at 4°C to remove exogenous EVs. The cultures were grown for 36 h using a gas mixture of 5% O2, 5% CO2, and 90% N2 (Lawres et al., 2016). In our hands, 1N ring parasites grew to 2N, 4N, and >4N, but the parasitemia of the culture did not increase. No new cycles of invasion occur, resulting in the same parasitemia as found *in vivo*, although culture medium had higher numbers of EVs than *in vivo* because of the higher parasite load within each RBC. Post-incubation, these culture supernatants were used to purify EVs as detailed below.

### Isolation and staining of extracellular vesicles by differential ultracentrifugation from *B. divergens* and *B. microti* culture system

EVs were purified from culture supernatant of uRBCs or iRBCs from both *B. divergens* and *B. microti* by sequential centrifugations at 1600 × g, 3600 × g, and 12,000 × g each for 15 min each at 4°C. The supernatant was then filtered with 0.2-μm filter to remove any cellular debris and then spun at 100,000 × g for 2 hr at 4°C in Beckman Coulter SW28 tube to get a pellet enriched in EVs. This was resuspended in serum-free RPMI, layered on top of 8 ml 60% sucrose, and centrifuged for 16 h at 4°C in a Beckman SW41 tube at 100 000 × g. Purified EVs were collected from interface (500 ml) and washed with 10 ml phosphate-buffered saline (PBS) by spinning at 100,000 × g for 1.5 h at 4°C in a Beckman Coulter SW41 tube.

For carboxyfluorescein succinimidyl ester (CFSE) staining of EVs, 500 μl CFSE (V12883 Invitrogen) was added at a final concentration of 10 μM. The labeling was done for 15 min at 37°C, following which 30% bovine serum albumin (BSA) stock (Sigma-Aldrich, St. Louis, MO) was added at a final concentration of 1% BSA to stop the labeling. CFSE-labeled EV pellet was layered on 60% sucrose, spun for 16 h in a Beckman SW41 tube at 100 000 × g. EVs collected from interface were washed with 8 ml PBS by spinning 1.5 h in a Beckman SW41 tube at 100 000 × g.

### Flow cytometry-based confirmation of identity of extracellular vesicles

As recommended by the International Society for Extracellular Vesicles, following differential centrifugation, we verified the identity of EVs isolated from uRBCs and iRBCs using standard markers, as previously described (Andreu and Yanez-Mo, 2014). Toward this, we used Exosome-Human CD9 and Exosome-Human CD81 Flow Detection Reagents (Invitrogen), which contained magnetic beads attached to capture antibodies CD9 and CD81, respectively. The enriched fraction of EVs was incubated with the magnetic beads, with appropriate controls, as outlined by the manufacturer. Following this, two detection antibodies CD9 and CD81 conjugated to PE fluorophore (BD Pharminogen) were used at manufacturer-directed dilutions. The samples were read in the Cytoflex by Beckman Coulter using the violet side scatter and Phycoerythrin (PE) channels.

### Flow cytometry–based calculation of parasitemia

Parasitemia measurements for *B. divergens* and *B. microti* were done using previously established protocols in our lab (Cursino-Santos et al., 2017). Briefly, mouse erythrocytes (1 × 107 cells/ml) were identified by allophycocyanin (APC) rat anti-mouse TER-119 at a final concentration 0.005 μM (BD Pharmingen). iRBCs were identified by staining parasite DNA using Hoescht 33342 (0.1 μM final concentration; Thermo Fisher Scientific). As all RBCs lack a nucleus, RBCs with a positive signal for DNA represent infected host cells bearing parasites. For *in vitro* cultures of *B. divergens* in human blood, samples were stained with the DNA-dye Vybrant® DyeCycle™ Green (1:500) and BV421 mouse anti-human CD235a (BD-562938; 1:500), which labels human RBCs. Samples were analyzed on an LSR Fortessa SORP analyzer (BD Biosciences), equipped with a 355-nm UV laser for Hoechst detection (361/486 nm), a 640-nm red laser for APC–TER-119 detection [650/60 nm bandpass (BP)], a 488-nm blue laser for Vybrant® DyeCycle™ Green detection (530/30 nm BP), and a 405-nm violet laser for anti-GPA detection (450/50 nm BP) in 10,000 target events (iRBCs). The forward scatter threshold was set on 300, and 10,000 total events were acquired at “low” flow rate. FACSDiva software (version 6.2; BD Biosciences) was used for data analysis. All parameters were processed using log scaling.

### MicroRNA seq and microarray

Up to 12 T-75 culture flasks of uninfected RBCs (uRBCs) or *B. divergens*–infected RBCs (iRBCs) were used. EV pellet, obtained after from differential centrifugation and 60% sucrose cushion, was washed with PBS and stored at −70°C until ready to be shipped to Norgen Biotek (Thorold, ON, Canada), where miRNA isolation, concentration, and quality check were performed. The samples were treated with RNase prior to RNA isolation to remove extravesicular RNA. RNA from EVs was isolated using the Norgen’s Plasma/Serum RNA Purification Mini Kit (Cat. 55000) according to the manufacturer’s instructions (Norgen Biotek, Thorold, ON, Canada). Quantification of isolated RNA using RiboGreen assay was determined along with reverse transcription quantitative real-time PCR amplification of the 5S rRNA and miR-21 to indicate the quality RNA. Norgen Biotech shipped the purified miRNA to LS Sciences (Houston, TX), where miRNA sequencing and miRNA microarray were performed and analyzed. LS Sciences human miRNA array used Part No. MRA-1001B2, version number miRHuman_21, and this was based on Sanger miRBase Release 21 (http://www.mirbase.org/). Detailed methods are provided in **Supplementary File 3**.

### Electron microscopy of extracellular vesicles and of extracellular vesicles with red blood cells

Sucrose-purified EVs or iRBCs were washed with 1× PBS and resuspended in fixative with 1% paraformaldehyde and 0.1% glutaraldehyde in 0.1 M cacodylate buffer for 1 h at 4°C, washed in 0.1 M buffer (pH 7.4). They were then treated with 50 mM ammonium chloride to quench the remaining aldehydes and spun for 1.5 h in a Beckman SW41 tube at 100,000 × g and resuspended in 50 ml PBS. Negative staining of purified vesicles from a sucrose gradient interface was performed by using uranyl acetate (1%) in water. After sections were stained with uranyl acetate, they were observed using a Philips 410 electron microscope (Holland).

### Imaging flow cytometry antibody staining and acquiring

The 100,000 g EV-enriched pellet obtained after differential centrifugation was washed with PBS by spinning 1.5 h in a Beckman SW41 tube at 100 000 × g, resuspended in 500 μl PBS. Staining for *B. divergens* iRBC–derived CFSE-labeled EVs was done as follows: 2 μl rabbit anti-Bd37 antibody (used at 1:200) in 1% BSA/PBS at 4°C for 1 h, followed by a wash step and addition of 4 μl Texas Red® (TR) goat anti-rabbit IgG antibody at 1:100 in 1% BSA/PBS (Vector Laboratories, Inc., Burlingame, CA). Antibodies prior to use were always spun down and filtered with a 0.22-μm syringe filter to get rid of any aggregate. *B. microti*–derived CFSE labeled EVs were labeled similarly. Primary antibody anti-BM2 was used at 1:125, and secondary antibody Texas Red® horse anti-mouse IgG antibody (Vector Laboratories, Inc., Burlingame, CA) was used at 1:100.

For the internalization experiment involving RBCs, CFSE-labeled EVs were obtained as outlined above. BCA protein analysis was done, and 50 μg of EVs was incubated with infected parasite culture at 5% hematocrit and 35% parasitemia in 100 μl of complete RPMI. At 1 and 3 h, cells were washed twice with RPMI to remove unbound EVs. This was followed by staining with primary antibody against red cell marker Band3 (1:250), which was conjugated to a secondary antibody linked to the APC fluorophore (Hu et al., 2013). In addition, DNA dye Hoechst was used at 0.1 μM, and the samples were incubated at room temperature for 30 min. Cells were washed twice and were immediately run on the ImageStream.

For internalization experiment using monocytes, human monocytes were purified using anti-human CD14 microbeads (Miltenyi Biotec, Auburn, CA) (purity > 95%), as previously reported by our group (Liu et al., 2019). Purified EV were labeled with CFSE, as elaborated above. CFSE-labeled EVs were co-cultured with purified monocytes (2 × 105/well) in 96-well plates containing RPMI-1640 medium supplemented with 100 U/ml penicillin, 100 µg/ml streptomycin, and 10% heat-inactivated fetal bovine serum (Thermo Scientific) overnight. For analysis, Accutase (Sigma-Aldrich, St. Louis, MO) was added to the 96-well plates, following manufacturer’s instruction to detach all cells in the wells. Following two washes, anti-human CD45-APC (HI30 from BD Biosciences) was used at manufacturer’s standardized dilution.

Images were acquired using a 12-channel Amnis ImageStreamX Mark II (Luminex, Austin, Texas). Imaging flow cytometry (IFC) samples were acquired at 60× magnification on low speed and excitation lasers 488 (Channel 2 CFSE) and 562 (Channel 10 for Texas Red). Brightfield images were acquired in Channels 1 and 9, whereas side scatter was acquired in Channel 6. Speedbead Kit Amnis® Catalog #400041 were used. For RBC internalization experiments, CFSE-labeled EVs were incubated with red cells for the mentioned time points. RBCs were stained using an antibody against Band3 conjugated to secondary antibody linked to APC and acquired using a 640-nm laser (Channel 11 for APC). In addition, Hoechst was used to label parasite DNA and was acquired using a 405-nm excitation laser (Channel 7 for violet). For monocyte internalization experiment, 40× magnification was used. Compensation (.cif) files were applied to all the raw data files (.rif) to obtain data files (.daf) that were further analyzed in IDEAS® (data analysis software of Amnis Imagestream) 7.1 or FCS Express (Image) Version 7.1 to obtain flow plots and statistics. Internalization Wizard of the IDEAS® software was applied, as previously described (Phanse et al., 2012). Out of focus cells and doublets were removed from analysis by appropriate gating (shown in **Supplementary Figure 2**). Cell boundary was defined by Band3 staining, and internalization of CFSE-labeled EVs was probed. The analysis was applied on all single cells in the population, and a histogram was obtained. A population of cells of more than zero were labeled as “internalized,” and those below 1 were “not internalized.” Values of the internalization score were calculated for at least 10,000 cells.

### Immunoblotting of extracellular vesicles

Sera of blood donors screened positive for *B. microti* were collected as per the guidelines of New York Blood Center Institutional Review Board (n = 3). Immunofluorescence was used to determine the dilution at which these sera recognized *B. microti*–infected cells. Mice were injected with *B. microti*, as explained above (n = 5), and reactivity of their sera was monitored over time. The above human sera and mouse sera were then used to detect immunoreactivity of purified EVs derived from *B. microti*. Purified EVs were incubated with 0.25% (w/v) trypsin solution at 37°C for 30 min, which results in digestion of non-associated membrane proteins, based on a previous protocol (Saari et al., 2015). Protease inhibitor was added and lysed in Laemmli sample buffer (Bio-Rad). Equal amounts of protein from uninfected and *B. divergens*– or *B. microti*–derived EVs were loaded onto 4%–20% Mini-PROTEAN TGX™ gels (Bio-Rad). After electrophoresis, the proteins were transferred onto nitrocellulose membrane (Bio-Rad). The membranes were blocked with 5% (w/v) skim milk powder in PBS Tween 20 and then incubated for 1 h at room temperature with 5% milk in PBS Tween 20 with primary antibody *B. microti*–infected human or mice sera, as needed. This was followed by horseradish peroxidase (HRP)–labeled secondary antibody (Amersham ECL mouse IgG, HRP-linked whole antibody and Amersham ECL rabbit IgG, HRP-linked whole antibody, GE Healthcare; anti-human IgG (H+L) antibody, peroxidase-labeled, Kirkegaard & Perry Laboratories, Inc.; or donkey anti-goat IgG antibody, HRP conjugate, Sigma-Aldrich, St. Louis, MO) diluted in 5% non-fat milk.

## Results

### A heterogeneous population of extracellular vesicles are released by both *B. divergens*–infected red blood cells in culture and in circulation of *B. microti*–infected mice

**Figure 1:**
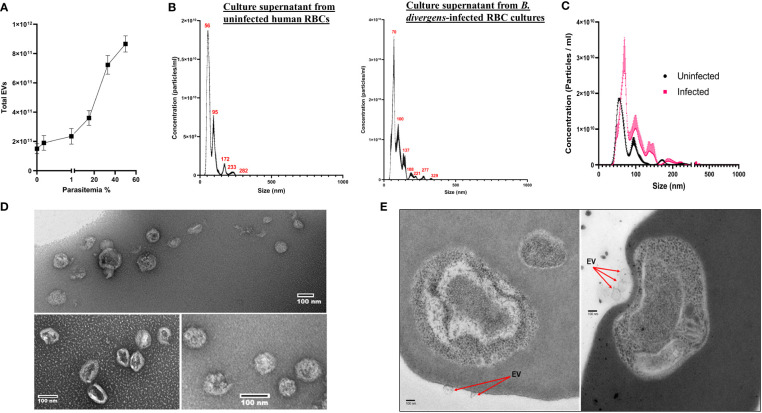
Characterization of extracellular vesicle (EV) derived from *B. divergens*–infected red blood cells (RBCs). EVs present in spent culture media of uninfected RBCs (uRBCs) and *B. divergens*–infected RBCs (iRBCs) were used for the analysis. **(A)** Total EVs in *B. divergens* spent culture supernatant increase with an increase in parasitemia. Approximately, a 4-fold increase in EV numbers was obtained as parasitemia increased from 10% to 60%. **(B)** Nanoparticle tracking analysis was performed on supernatant from uRBCs and *B. divergens* iRBCs. Profile of size (in nm) concentration is shown. As evident, the highest concentration peak of EVs in uRBCs was ~55 nm, whereas for iRBCs, it was ~70 nm. iRBCs also demonstrated a wider distribution of size of EVs as compared with uRBCs. **(C)** Direct comparison between uRBCs and iRBCs (~10% parasitemia) with respect to size (in nm) of EVs. At this parasitemia, iRBCs had 1.8- to 2-fold higher number of EVs as demonstrated by “concentration (particles/ml)” in the y-axis. **(D)** Density gradient–dependent purification of EVs was performed, followed by negative staining and visualization under transmission electron microscopy. A range of different sizes of EVs from *B. divergens*–derived culture supernatant were seen, concurrent with nanoparticle tracking analysis shown in **(B)**. **(E)** When RBCs were visualized, particles in the size range of EVs were seen on the surface of red cells shown in **(C)**.

**Figure 2:**
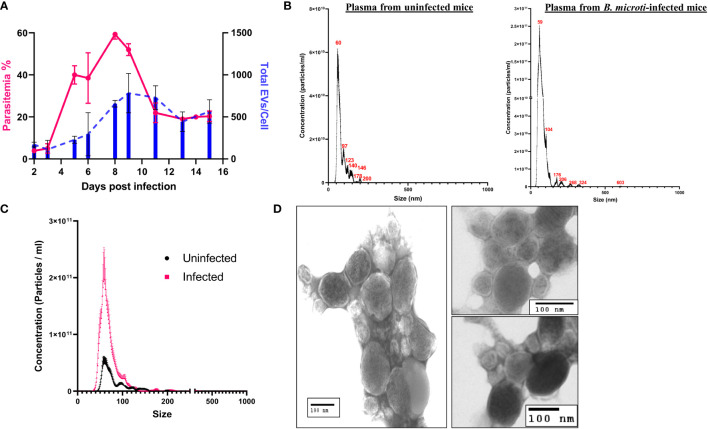
Characterization of extracellular vesicle (EV) derived from the plasma of control uninfected and *B. microti*–infected mice. EVs present in the plasma of uninfected and *B. microti*–infected mice were used for the analysis. **(A)** Comparison of total EVs/red cell (blue) with parasitemia (pink) and days post infection (x-axis) reveals that as parasitemia increases, there is an increase in the number of EVs, which peaks with a peak in parasitemia. As parasitemia begins to fall around day 12, the number of EVs/RBCs also decreases (n = 3 mice). **(B)** Nanoparticle tracking analysis in uninfected and *B. microti*–infected mouse plasma reveals the size distribution of EVs. As evident, most of the EVs in both sample sets were in the range of 60–120 nm. iRBCs showed a wider distribution of size of EVs. **(C)** A direct comparison between EVs from uRBCs and iRBCs (day 6 post invasion) in the mouse model. As evident, iRBCs showed a ~5-fold increase in EVs as compared with plasma from uninfected mouse. **(D)** EVs were enriched from mice infected with *B. microti* using the protocol detailed under Methods and visualized using transmission electron microscopy. A heterogeneous population of EVs with respect to size was shown, which is concurrent with the nanoparticle tracking analysis shown in **(B)**.

To assess the presence of EVs in Babesia, we used two different infection model systems, using two distinct Babesia species, *B. divergens* and *B. microti*. The study of EVs in the context of hemoparasite infection is complicated by the fact that the host RBC makes and releases vesicles into the extracellular environment, necessitating a uRBC control in all experiments. As *B. divergens* is easily cultured in human RBCs *in vitro*, we used culture supernatants from uRBC and iRBCs as the source of EVs from this parasite. *B. microti*, on the other hand, requires an animal model to establish infection. C57/Bl6 mice were infected with *B. microti*, and plasma from uninfected and infected mice were analyzed for EVs. To determine the size range heterogeneity and the concentration of EVs in *B. divergens* culture supernatant, we used the nanoparticle tracking analysis technology offered by NanoSight™, which quantifies particles between 0.01 and 1 μm in small volumes (10–20 μl) of culture supernatant/plasma. For each set, a total of three samples were analyzed. First, we determined the total EVs in spent culture supernatant from increasing parasitemia percentage of parasitized host RBCs (cultures of *B. divergens* iRBCs). As shown in **Figure 1A**, we observed that as parasitemia increased, the total number of EVs in the conditioned medium of *B. divergens*–infected cells showed a proportionate increase. At 50% parasitemia, the EV concentration was almost four times that obtained from a 10% parasitemia culture, representing a 16-fold increase in EV output over uRBCs. This suggests that infection results in an amplification of EV yield in the spent media to further examine the sizes of the particles from uRBCs and iRBCs’ spent media; culture supernatant originating from four distinct ~10% parasitemia asynchronous cultures were used as the source of EVs in these analyses. The light scattering data analysis revealed particles ranging from size 50 to 500 nm, with the modal size of 66.5 ± 2.5 nm, with peaks seen at 100, 137, 188, 221, 277, and 329 nm. EV preparation from uRBCs that were kept under identical culture conditions for the same amount of time yielded a slightly different mode of size range (56.1 ± 0.9 nm). **Figure 1B** shows a representative graph of concentration (particles/ml culture supernatant) versus size of the EV in nm. As evident, the highest concentration of EVs was in the range of 70–120 nm. There was a longer distribution of size of EVs along the x-axis (**Figure 2A**) in iRBCs as compared with uRBCs. Furthermore, as shown in **Figure 1C**, concentration of EVs from iRBC conditioned media was ~2–3 times higher than EVs purified from uRBCs. This establishes that Babesia iRBCs secrete a much higher number of EVs as compared with uRBCs. To further study the Babesia EVs, they needed to be purified from culture supernatant. As described in detail under Methods, a method was developed based on previous literature (Mantel et al., 2013; Mantel and Marti, 2014). Briefly, the supernatant/plasma were spun at 100,000g for 2 h, following which it was laid on a continuous sucrose gradient and spun for 15 h at 100,000g. The interface was collected and used as the source of EVs for all described studies. Furthermore, the identity of EVs in this fraction was confirmed using standard markers (CD9 and CD81), as detailed under Methods and **Supplementary File 4**. Furthermore, to assess the purity and size of the EVs obtained from the gradient, we stained the isolated EVs using negative stain methodology, followed by transmission electron microscopy (TEM). Our analysis (**Figure 1D**) revealed the approximate size and morphology of EVs, concurring with the nanoparticle tracking analysis data and those obtained in other parasite systems (Marti and Johnson, 2016; Moyano et al., 2019; Sharma et al., 2020). Examination of thin sections of *B. divergens* iRBCs using TEM revealed a population of small vesicles budding from the membrane of iRBCs (**Figure 1E**), indicating a possible interaction of EVs with iRBCs, as elaborated in the following section.

To test if *in vivo* Babesia infection also results in similar EV release dynamics, we infected mice with *B. microti* and monitored plasma from control and infected mice for the presence and frequency of EVs using the service offered by NanoSight™ (**Figure 2**). A previous study in *B. microti* demonstrated the presence of vesicular-mediated antigen export (Thekkiniath et al., 2019), but to the best of our knowledge, no report of characterization of these EVs is available. First, we examined the total EVs in infected versus uninfected mice plasma (n = 3) up to 16 days post infection. As infection results in loss of hematocrit, the EV numbers were normalized to the total number of red cells. As shown in **Figure 2A**, the total number of EVs in the plasma of infected mice increased with progression of parasitemia. As evident, parasitemia reaches a maximum of 59.33 ± 1.33% on day 8, whereas the total number of EVs/cell reaches a maximum of 783 ± 165.5 on day 9 post invasion. A size versus concentration analysis of EVs from uninfected and infected plasma (n = 3; day 6 post invasion) revealed key differences (**Figure 2B**); whereas uninfected plasma exhibited peaks 60, 97, 123, 140, 146, 179, and 200 nm (mode of 65.6 ± 3.0 nm), the peaks for EVs from the plasma of infected sample were at 59, 104, 176, 206, 268, 324, and 603 nm (mode of 57.5 ± 3.2 nm). A direct comparison between plasma from uninfected and infected mice (**Figure 2C**) shows that plasma from infected mice have 3- to 4-folds higher EVs as compared with plasma from uninfected mice. Furthermore, purification of EVs was performed using plasma from infected mice, following a short *ex vivo* culture of *B. microti*, as detailed under Methods. TEM analysis (**Figure 2D**) shows that the RBCs from infected mice release EVs *in vivo*, and the size and shape of these vesicles are consistent with EV descriptions from other parasites (Marti and Johnson, 2016; Babatunde et al., 2018). It is important to emphasize that EVs from both host and parasite are enumerated in such analyses.

These results (**Figures 1**, **2**) demonstrate the presence of EVs in both *in vitro* and *in vivo* Babesia infection of host RBCs. Although uRBCs in both models release EVs, we show that infection results in a significantly higher number of EVs. Furthermore, this EV frequency is proportional to the parasite load in both the *in vitro* and *in vivo* models of Babesia–host RBC infection.

### Purified extracellular vesicles can be internalized by red blood cells and immune cells in culture

**Figure 3:**
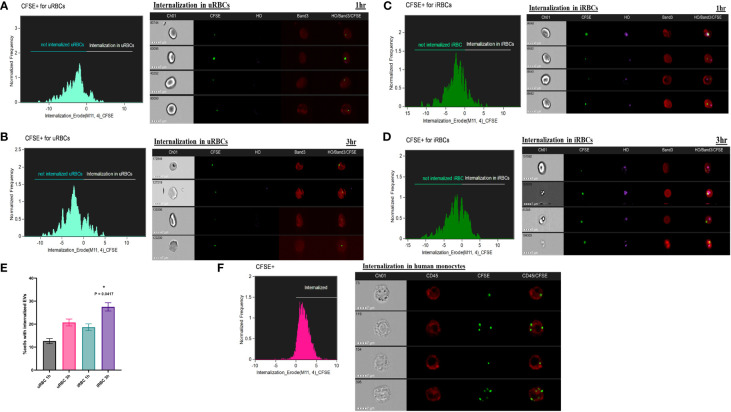
Internalization of carboxyfluorescein succinimidyl ester (CFSE)–labeled extracellular vesicles (EVs) by red blood cells (RBCs) and monocytes. (a–e) 50 μg of purified EVs from spent culture supernatant of *B. divergens*–infected RBCs (iRBCs) were CFSE (green) labeled. These were incubated with iRBCs at 35% parasitemia for 1 and 3 h and stained with Band3-APC (red) and Hoechst (pink). ImageStream was performed followed by the application of Internalization Wizard of IDEAS™ software. In the histogram obtained, cells above zero are “internalized,” whereas those below are “not internalized.” Gating strategy is elaborated in **Supplementary Figure 2** under **Supplementary File 4**. **(A, B)** The histograms show internalization of CFSE-EVs in uninfected RBCs (uRBCs) (Band3+, HO−) at 1 and 3 h, respectively. Representative images of internalized EVs are shown. **(C,D)** The histograms show internalization of CFSE-EVs in iRBCs (Band3+, HO+) at 1 and 3 h, respectively. Representative images of internalized EVs are shown. **(E)** Quantification of percent cells with internalized EVs reveals that a marginally higher percentage of iRBCs internalize EVs than uRBCs (~1.5 folds). With time, internalization increases in both uRBCs and iRBCs [p = 0.0417 (*), n = 2]. **(F)** Monocytes were purified as elaborated under Methods and co-incubated with CFSE-labeled EVs. Internalization profile shows that >90% monocytes (labeled with CD45-APC in red) internalized CFSE (green)–labeled EVs purified from *B. divergens* spent culture supernatant.

We next examined if the purified EVs could be internalized by uRBCs and iRBCs, as has been previously suggested in other systems (Mantel et al., 2013; Marti and Johnson, 2016). IFC can be used to detect multiple fluorescent markers and, together with data on cellular and vesicle morphology, allowed us to study the localization and other specific characteristics of EVs in the context of the parasite. The ability to numerically score large numbers of acquired images is ideally suited to the analysis of internalization, and therefore, this approach was used. Multiple fluorescence tags were used in this analysis: RBCs were labeled with Band3-APC (Red), parasites were labeled with DNA dye Hoechst (pink), and purified EVs (50 μg) were labeled with Vybrant-CFSE (Green) and incubated with a culture at 35% parasitemia for 1 and 3 h, as detailed under Methods. Toward quantitation of percent CFSE+ cells, which were internalized, we used the “Internalization” feature in the IDEAS® statistical analysis software of the ImageStream, as elaborated under Methods. The internalization histogram divides cells that are “internalized” and “not internalized” as shown by values of internalization erode above 0 and below 0, respectively, on the x-axis. The number in the box of Channel 1 represents the unique serial number of the cell in focus for that particular sample. As shown in **Figures 3A–D**, EVs were seen internalized into both uRBCs and iRBCs at 1 and 3 h post co-incubation. Representative images of internalized CFSE-stained EVs are shown in all cases, and EVs were seen to be often co-localizing with the parasite. Quantitation was performed using a minimum of 10,000 cells. As shown in **Figure 3E**, the number of cells with internalized EVs was higher in iRBCs as compared with uRBCs. In addition, with an increase in time of co-incubation, an increasing percentage of cells (both uRBCs and iRBCs) were observed to uptake EVs. At 1 and 3 h, the percentages of uRBCs with internalized EVs ± SEM were 12.6 ± 1.1% and 20.70 ± 1.212%, respectively, whereas for iRBCs, they were 20.70 ± 1.5% and 27.50 ± 1.8%, respectively (n = 2, one-way ANOVA, p = 0.0417). Therefore, these results suggest that EVs have the potential of transferring cargo containing effector molecules to RBCs and thus mediate communication between both host and parasite and within parasite populations.

Next, we wanted to examine if immune cells, like monocytes, can take up EVs released into Babesia-infected culture supernatants. Toward this, we purified human monocytes using anti-CD14 microbeads and overnight (~12 h) co-incubated 50 μg of CFSE-labeled EVs with 2 × 105 purified monocytes. Cells were stained with CD45, a common marker of leucocytes. Images were analyzed on IDEAS, and internalization was calculated. Interestingly, a significant majority of monocytes (>90%) were able to uptake *B. divergens* spent media–derived EVs. An earlier report has shown uptake of Plasmodium falciparum–derived EVs into peripheral blood mononuclear cell activation (Mantel et al., 2013) and implicated in the “cytokine storm” associated with malaria (Marti and Johnson, 2016). Our current data imply a possible role of *B. divergens* iRBCs–derived EVs in activation of immune cells and require further functional characterization.

### Protein cargo from extracellular vesicles from *in vitro* and *in vivo* Babesia models are recognized by Babesia-infected human and mice sera

As EVs were released in the plasma of infected individuals, we hypothesized that their constituent proteins may be the target of the host immune response. To further investigate this, we used sera from both infected human and mouse hosts.

**Figure 4:**
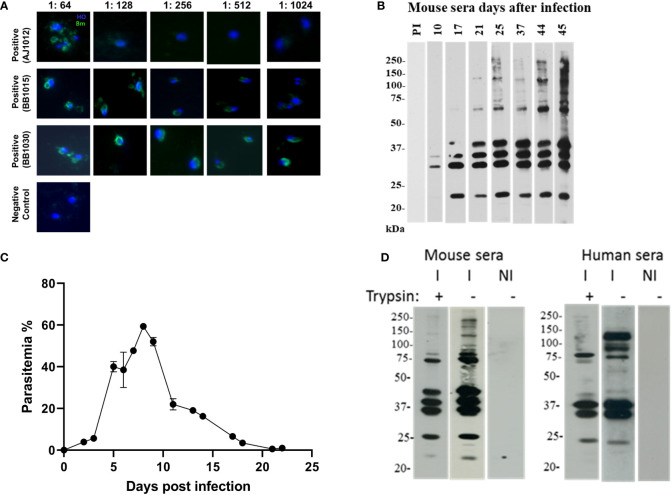
EV proteins are recognized by immune sera of *B. microti*–infected humans and mice. (a and b) Selection of sera and appropriate dilution to be used in extracellular vesicle (EV) immunoblots. **(A)** Sera were collected from human patient donors, and immunofluorescence was performed using *B. microti*–infected cells stained with Hoechst (blue). Anti-human secondary antibody (green) was used to detect immunopositive antigens. Three sera were used as labeled, and different sera titers were used. These sera were pooled and further used at 1:128 dilution. **(B)** Mice (n = 5) were infected with *B. microti*, and the reactivity of each mouse serum was monitored over days of infection. As shown, pre-immune (PI) sera did not react with *B. microti* lysate. At day 45, the maximum number of parasite antigens were recognized by the immune serum, and this was used for subsequent experiments. **(C)** Parasitemia profile of *B. microti*–infected mice is shown until day 22 post invasion. As evident, parasitemia increases and peaks around day 7, after which it progressively falls as the parasite is cleared by the immune system. By day 20, parasitemia reaches almost zero, and no further increase is seen. **(D)** EVs purified from control uninfected and *B. microti*–infected mice plasma (same number of EVs were used in all lanes) were run on SDS-PAGE and probed with immune human or immune mouse sera from **(A)** and **(B)**, respectively. NI refers EVs purified from naïve mouse, and these did not react with either sera. As shown in the “I−” lanes, several antigens were recognized by the immune human and mouse sera. In lanes “I+,” EVs were subjected to trypsin before running on the SDS-PAGE and then probed using the human and mouse sera. Several bands disappeared on this treatment, suggesting the presence of these proteins on the surface of the EVs.

Sera were selected from human donors who were immunopositive against *B. microti* in an Indirect immunofluorescence assay (IFA) analysis (**Figure 4A**). Three positive donor sera were chosen with titers of 1:64 (AJ102), 1:512 (BB1015), and 1: 1024 (BB1030). These human sera were pooled and used to probe the EV blots (**Figure 4D**, right panel). Mice sera were obtained from *B. microti*–infected C57/Bl6 mice. Mice were bled, and sera were analyzed for reactivity against *B. microti* lysates (**Figure 4D**). As shown, beginning from day 17, infected mice sera recognized multiple antigens on the lysate, and this peaked on day 45. Thus, day 45 sera from n = 5 mice were pooled and used to probe EV blots (**Figure 4D**, left panel). Equal amounts of purified EVs were loaded in all the lanes. **Figure 4C** shows the parasitemia profile of *B. microti* in mice. As evident, parasitemia increases initially and peaks at day 8 post invasion. Thereafter, the parasite is cleared by the immune system, and by day 20, parasitemia is almost zero with no further increase.

EVs were purified from both uninfected and *B. microti*–infected mice short-term *ex vivo* cultures (Lawres et al., 2016) lysed, and proteins were run on SDS-PAGE and blotted and probed with the various mice and human sera defined above. We were specifically interested in defining the surface proteome of the EVs since these proteins would be most exposed to the host immune system. Thus, to establish if they were EV surface or internal proteins, fractions containing purified vesicles were digested with either trypsin to ensure that proteins that are associated with the EV surfaces are stripped. We carried out Western blot analysis on purified EVs from only *B. microti in vivo* infections, as shown in **Figure 4D**. This is because *B. divergens* has no animal model system and there was no access to *B. divergens*–infected human sera. Immunoblotting analysis (**Figure 3C**) reveals presence of multiple *B. microti* proteins that are recognized by both infected mouse sera and immune-positive human sera (lanes I in each panel). When the EVs were treated with trypsin, multiple protein bands disappeared, suggesting that these were on the surface of the EV (lane I+ from each panel). We also examined reactivity of EVs from uninfected mice as controls, using a similar number of EVs and normalized for the same protein content. Lane NI contains EVs from naive mouse and does not react with either infected mouse/human sera, demonstrating the specific presence of parasite proteins only on EVs from infected mice. Thus, our results suggest that parasite-specific proteins are present both on the surface of EVs and within EVs derived from *B. microti*–infected mice, and importantly, these are recognized by the immune sera derived from both Babesia-infected humans and mice.

### Imaging flow cytometry–based confirmation and quantitation of parasite-specific extracellular vesicles derived from infected cells

**Figure 5:**
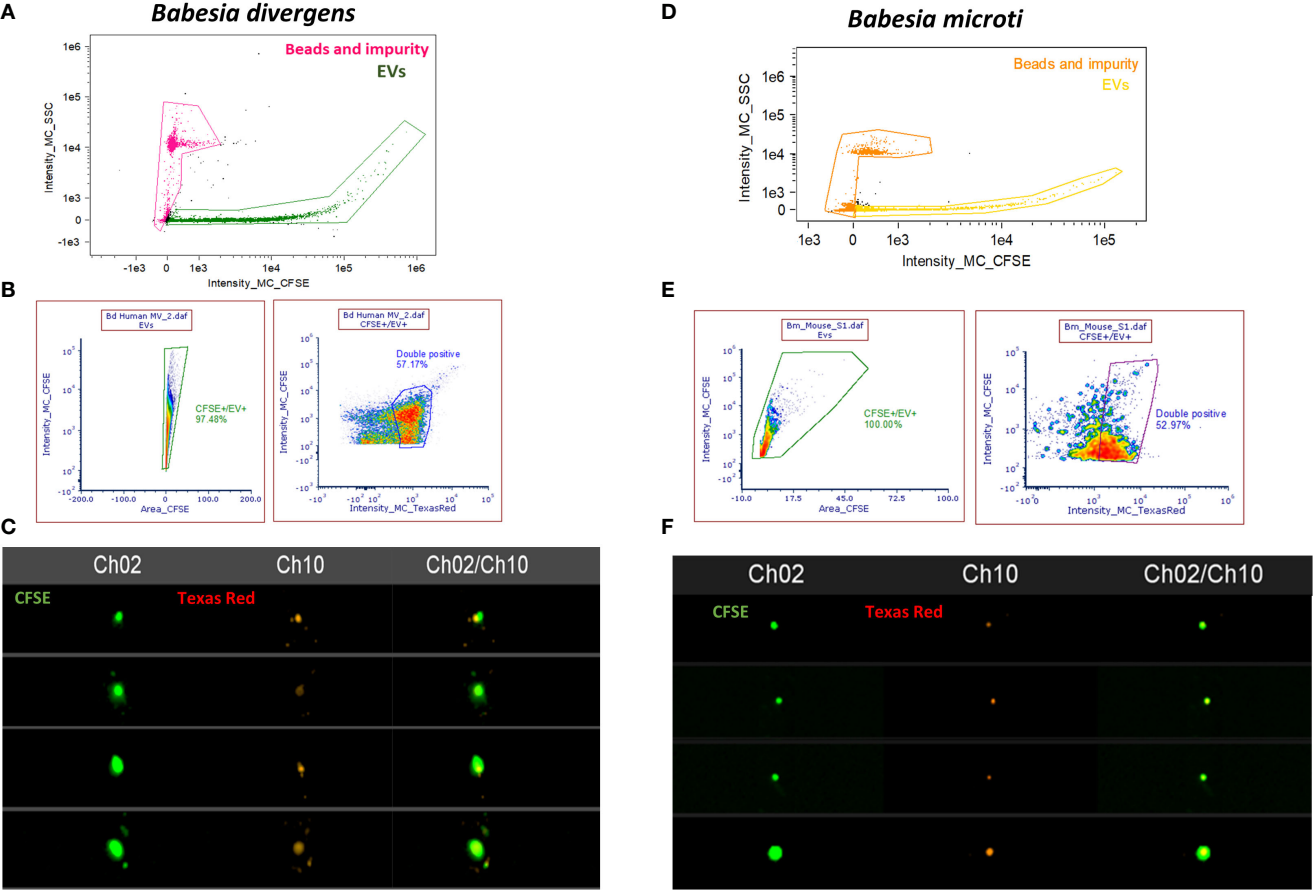
Imaging flow cytometry (IFC)–based quantitation of parasite-specific extracellular vesicles (EVs). **(A)** Gating strategy to select for EVs from *B. divergens*–infected cells. Debris and beads were excluded from further analysis. The EVs were stained with carboxyfluorescein succinimidyl ester (CFSE) (green) and Texas Red-Bd37, which is a *B. divergens*–specific marker (red). **(B)** CFSE-positive cells were gated based on the area-versus-intensity plot. Right panel shows events that were positive for both CFSE and Texas Red-Bd37 and represent ~60% of EVs. **(C)** Images showing EVs that were labeled with both CFSE (Channel 2) and TR (Channel 10) and the merged image for the two channels. **(D)** Similar gating strategy as **(A)** was used to exclude beads and debris from EVs derived from *B. microti*–infected plasma. **(E)** EVs were stained with CFSE (green) and TR-Bm2 (red), which is a specific marker of *B. microti*. CFSE+ cells were gated based on the area-versus-intensity plot. Right panel shows the events that were double positive for both (~50%). **(F)** IFC images showing EVs stained with CFSE (Channel 2) and TR (Channel 10) and their merged image.

For erythrocytic parasites, analysis of EVs was complicated because uRBCs also release vesicles. To obtain an idea of the proportion of EVs that are sourced from Babesia infection, we decided to use parasite markers to differentiate between EVs from uninfected versus iRBCs in both *B. divergens* and *B. microti* infection models. To identify *B. divergens*–specific EVs, we used antibodies against Bd37, which is an abundant 37-kDa protein from *B. divergens* (Delbecq, 2022). To identify *B. microti*–specific EVs, we used antibodies against BMN-2, which is an abundant protein from *B. microti* (Homer et al., 2000) (Elton et al., 2019). EVs were purified from ~30% to 40% parasitemia *B. divergens*–infected cultures and labeled with Vybrant-CFSE (Green) and Texas Red-Bd37. **Figures 5A, B** show the gating strategy used for EVs as a function of the intensity of side scatter signal and the intensity of CFSE signal and were labeled as EV+. Next, we plotted the intensity of CFSE signal versus the area of CFSE signal and gated cells that were labeled CFSE+/EV+. Unstained samples were run to determine the positioning of the gate. More than 90% of our EVs were labeled with CFSE. Next, we used this population and plotted the intensity of Texas Red versus the intensity of CFSE and labeled the positive population as “double positive” (**Figure 5B**). We found that ~60% of EVs were positive for both CFSE and Texas Red (TR). Thus, ~60% of EVs derived from infected cells’ conditioned medium contained the Bd37 marker, suggesting that they had been derived from parasite-infected host cells. Similarly, IFC analysis was performed for EVs purified from sera of *B. microti*–infected mice, where EVs were labeled with CFSE (Green) and the *B. microti*–specific marker Bm2 was used coupled with Texas Red (Red). **Figure 5D** shows the gating strategy used to separate *B. microti* EVs from beads and debris (intensity of CFSE vs. intensity of side scatter) and was labeled as “EVs.” Next, the intensity of CFSE+ was plotted against the area of CFSE+ to obtain CFSE+ cells. More than 90% of EVs and this gate were labeled as “CFSE+/EV+.” Next, Texas Red-Bm2 was plotted with CFSE+/EV+ as parent gate, and we found that ~50% of EVs were positive for TR-Bm2. This gate was labeled as “double positive.” Thus, 50% of EVs derived from the plasma of *B. microti*–infected mice showed specificity for the parasite-specific marker Bm2. **Figures 5C, F** show representative IFC images of EVs derived from *B. divergens*–infected cells and *B. microti*–infected cells, respectively. As evident in the images, EVs were labeled with both CFSE and TR, evidencing their origin from parasitized host cells.

Thus, when coupled to fluorescence detection, IFC analysis is a powerful tool that can be used to analyze specific vesicles within heterogeneous populations. As evident, using Bd37 (**Figures 5A–C**) and BMN-2 (**Figures 5D–F**) as parasite EV-specific markers, we were able to show that ~60% of EVs from *B. divergens* culture stained with Bd37 whereas ~50% of *in vivo B. microti*–infected mice EVs contained BMN-2 marker on their surface. Therefore, this represents the specific proportion of EVs released by iRBCs within the total EV population. EVs from uninfected cells did not show positive staining for either antigen.

### MicroRNA sequencing analysis indicates multiple human miRNAs and novel miRNAs that are enriched in extracellular vesicles derived from *B. divergens*–infected red blood cells

To identify miRNAs in EVs from uRBCs and iRBCs (~35%–40% parasitemia), we purified EVs, as outlined under Methods. Two different platforms were used to analyze miRNA, next-generation sequencing (NGS) and microarray analysis, to overcome the inherent drawbacks associated with each. Mature miRNAs are very short and thus require a rather error-prone identification method. miRNAs share high-sequence homology within families, with as low as one base difference, which can be difficult to differentiate, and miRNAs are known to have many isoforms due to RNA editing, resulting in single-nucleotide polymorphisms. These factors often present as challenges for primer or probe design and hybridization in microarrays. In NGS, sequence similarity of miRNAs can present a problem in discriminating between miRNAs prone to sequencing errors. The short and variable length of miRNA further reduces the ability to accurately identify the border between the miRNA and the adaptor (Willenbrock et al., 2009). Therefore, we used two different analytical platforms.

**Figure 6:**
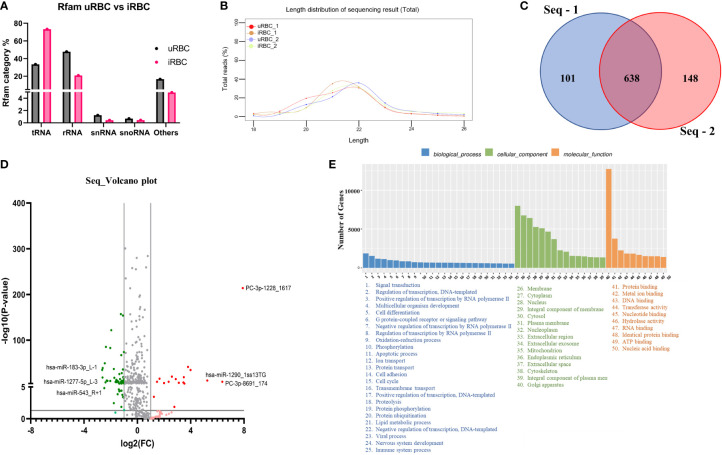
MicroRNA (miRNA) Illumina sequencing results in identification of differential miRNA expression in extracellular vesicles (EVs) from uninfected RBCs (uRBCs) and *B. divergens*–infected RBCs (iRBCs). **(A)** Percentage of different non-coding RNAs in EVs from uRBCs (black bars) and iRBCs (pink). “Others” refers to non-coding RNAs that did not match any known sequence in the Rfam 14.8 database. **(B)** Length distribution of small RNA used in the study was found to be between 20 and 24 nt. **(C)** Venn diagram depicting differences and similarities between the biological replicates used in the study shows significant overlap between the replicates. **(D)** Volcano plot showing differential expression of miRNAs. FC on x-axis refers to fold change in *B. divergens*–infected derived EVs compared with uRBCs and FC of more than/less than 2 were considered significant (shown by two vertical lines). Y-axis shows the statistical significance, and the cutoff chosen was p < 0.005, as shown by the black horizontal line. Gray dots were miRNAs that were non-significant in terms of p value and FC. Light red and light green dots represent miRNAs that were upregulated and downregulated, respectively, with an FC of more than/less than 2 but did not pass the p-value test. Dark red and dark green dots represent miRNAs with FC more than or less than 2, respectively, and significant p-values. The top three miRNAs in each set were identified. **(E)** Biological pathways (blue), cellular localization (green), and molecular function (orange) related to the miRNAs identified in the analysis and its related number of genes were plotted as bar graphs. As shown, several pathways are hypothesized to be altered by these miRNAs.

**Figure 6A** shows the analysis of miRNA population within the EVs using the NGS platform. The percentage of Rfam categories (Rfam refers to the collection of non-coding RNAs found in the sample) obtained from EVs of uRBCs and iRBCs are shown in **Figure 6A**. As evident, iRBCs (pink bars) had a higher content of tRNAs than uRBCs (black bars), and both had a miniscule percentage of snRNAs and snoRNAs. The small RNAs derived from the EVs were next analyzed to obtain the length distribution, and it was found that a majority of small RNAs were between 20 and 24 nucleotides, which is the size range for miRNAs (**Figure 6B**). The sequences obtained from the Illumina HiSeq were then mapped to miRbase 22.0. A total of 805 known miRNAs and 169 novel miRNAs, which have not previously been mapped in the database, were found in the analysis (Supplementary File 1). The criteria of secondary structures and annotations have been described in Supplementary File 1. The analysis was performed with two biological duplicates, and as shown in **Figure 6C**, they had 638 miRNAs in common among them. Next, we plotted the volcano plot in **Figure 6D** to visualize differentially expressed miRNAs in EVs derived from uRBCs and iRBCs. Gray dots represent the miRNAs that did not clear the statistical significance cutoff (p < 0.05) and/or had a fold change (FC) of expression in iRBCs/uRBCs < 2. Light green and light red dots represent the miRNAs that have an FC of < 2 and > 2, respectively, but do not pass the statistical significance test. Dark green and dark red dots are representative of miRNAs that have an iRBC/uRBC FC ≤ 2 or ≥ 2 and pass statistical significance test. The top 3 miRNA species from each set were marked. As shown in **Figure 5D**, the top three miRNAs identified in the analysis were novel miRNAs (labeled as PC) whose functions are yet unknown or have a 1-bp difference on the right (R) or left (L) from the annotated miRNA sequence. The gene ontology analysis of target genes identified several different pathways (**Figure 6E**) in which these miRNAs may be involved across different cellular locations. Thus, establishing miRNA signatures of both EVs from uRBCs and iRBCs and analyzing the differential expression between them can be used further to pin the functionality of these miRNAs and their possible role in cell-to-cell communication and disease pathology.

We next turned to another classical miRNA detection and analysis platform to determine if it could be used to discriminate between the RNA cargo of EVs released from uRBCs and iRBCs. Toward this, RNA was isolated from uRBCs and *B. divergens*–infected culture–derived EVs using Norgen’s Plasma/Serum RNA Purification Mini Kit (Cat. 55000) according to the manufacturer’s instructions and sent for hybridization and identification of miRNAs in these samples.

**Figure 7:**
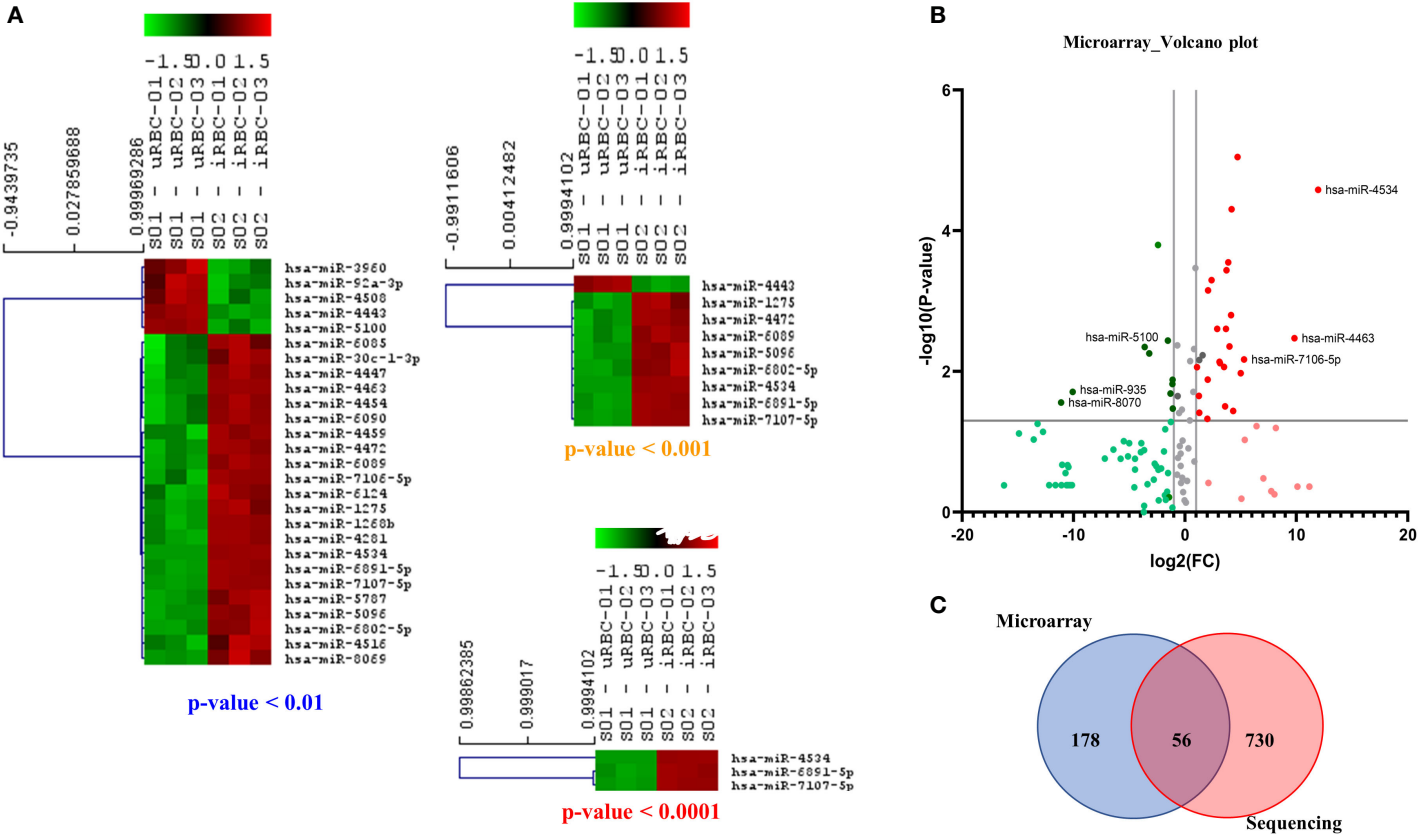
Microarray-based analysis of microRNAs (miRNAs) from extracellular vesicles (EVs) derived from uninfected RBCs (uRBCs) and *B. divergens*–infected RBCs (iRBCs). **(A)** Heat map shows the miRNAs that were changed at different *p*-value cutoffs (<0.01, <0.001, and <0.0001) between EVs from uRBCs and iRBCs (n = 3). As shown, these miRNAs were differentially expressed between the two sample sets. **(B)** Volcano plot representing the fold change (FC) and p-value of miRNAs differentially expressed in EVs from iRBCs with respect to those from uRBCs. Interpretation of colors is identical to **Figure 6D**. The top three upregulated and downregulated miRNAs in the analysis have been identified. **(C)** Venn diagram showing the overlap between miRNAs detected by microarray versus sequencing using next-generation sequencing. Fifty-six miRNAs were detected in both the platforms, whereas 178 and 730 unique miRNAs were detected by the two platforms, respectively.

A list of reporters used in the array is available in **Supplementary File 2**. As shown in **Figure 7A**, the miRNAs depicted in the heat map had statistically significant differences in expression levels in uRBC- versus iRBC-derived EVs using different p-value cutoffs. With a stringency of p < 0.0001, three miRNAs were observed to be differentially expressed between the two samples. In the heatmap, green depicts downregulation, whereas red depicts upregulation of miRNA expression. Of these, miR-4534 had been associated with cancer (Nip et al., 2016), whereas no data exist for the other two. To better visualize the different miRNA changes, we constructed the volcano plot with FC of iRBC/uRBC derived in the x-axis and statistical significance of = in the y-axis (p < 0.005 as seen in **Figure 7B**). The colors used in the plot **Figure 7B** have identical interpretation as **Figure 6D**. As shown, miR-4534, miR-4463, and miR-7106-5p were the ones with maximal upregulation. Interestingly, miR-4463 is associated with apoptosis and oxidative stress in endothelial cells. Elevated oxidative stress in the host cells has been linked to intracellular parasite pathogens like *Mycobacterium tuberculosis* (Chawla et al., 2012) and *P. falciparum* (Beri et al., 2017; Beri et al., 2019), and our previous work had shown that *B. divergens*–infected red cells experience disturbed redox homeostasis (Beri et al., 2022). Thus, miRNAs may play a role in causing parasite-related oxidative stress and could be investigated in future transduction experiments. Both our platforms detected an upregulation of miR-4454, which has been associated with severe thrombocytopenia in P. vivax–infected human plasma. Data in our microarray platform showed a 3.5-fold increase in miR-4497 in iRBC-derived EVs. These have been associated with splenic sequestration in *P. falciparum*; however, their role in *Babesia* infection is yet unknown. Furthermore, miR-5787 was found to be 15-fold elevated in iRBC-derived EVs as compared with its uninfected counterpart. miR-5787 has been implicated in inhibition of eIF5 in fibroblasts, but their significance in *Babesia* infection is yet unknown. As seen in **Figure 7C**, our NGS and microarray analysis had a significant overlap with differential expression observed in 56 miRNAs across both platforms. The NGS platform clearly was able to detect more miRNAs than microarray, which is limited by the probes used in the analysis. Overall, significant differences in the levels of multiple miRNAs between EVs from uRBCs and iRBCs were detected, and a few of them have been implicated in pathogenesis of other diseases including malaria, tuberculosis, and cancer.

## Discussion

EVs are generally categorized based on size and biogenesis, with exosomes ranging in size from 30 to 150 nm and MVs ranging from 150 nm to 1 μm (Colombo et al., 2014). Although the majority of the vesicles identified in our study were smaller than 150 nm (**Figures B**, **2B**), we have not purified the vesicle population based on size, and there is considerable overlay in both size and density between exosomes and MVs. Hence, because of the controversies and difficulties in defining and distinguishing between these two types of vesicles, we opted to use the more conservative term of EVs for the vesicles we have identified and characterized in this article.

In the last decade, EVs have been characterized in both unicellular and multicellular parasites, including apicomplexans, kinetoplastids, and parasitic worms, where they have been shown to mediate both host–parasite and parasite–parasite communication (Marti and Johnson, 2016). Our study is the first to document similar EV secretions from Babesia species, using both *in vitro* cultures and *in vivo* infection models, although a previous report (Thekkiniath et al., 2019) has documented evidence for vesicular-mediated antigen export in *B. microti* (Thekkiniath et al., 2019). Use of both species is critical because the mammalian circulation offers a different and dynamic environment from culture conditions; thus, *in vivo* derived EVs may differ in composition and/or activity from the culture-derived EVs. Like other related parasites, Babesia exploits dual hosts, requiring the ability to sense environmental changes and rapidly respond to such changes in terms of both regulating parasite population numbers and modulating host response to the infection (Cursino-Santos et al., 2016). Therefore, like other pathogens, Babesia must develop effective strategies to survive in a variety of environments—both supportive and hostile. EV communication may represent one such strategy that Babesia exploits to ensure successful propagation (Lobo et al., 2019). We show that EVs are in fact taken up by both uninfected and infected cells in culture through labeling and uptake experiments. Thus, EVs may mediate communication among parasite-infected cell populations, as has been shown for malaria (Mantel et al., 2013). Such communication could help parasites to sense population density and allow shifts among the parasite populations to ensure persistence (Lobo et al., 2019). An interesting study from *P. falciparum* cultures provides similar evidence of the EV-associated PfLDH regulation of parasite population by inducing apoptosis in highly parasitized cultures (Correa et al., 2019).

The characteristics and composition of EV populations from hemoparasites are highly heterogeneous, differing in subcellular origin and their processing route, being either RBC membrane or parasite membrane derived. This is reflected in our data (**Figures 1B**, **2B**) where we have identified such a heterogeneous population of EVs in both culture supernatants and infected mouse plasma in terms of both vesicular size and vesicular staining. NanoSight analysis demonstrated that the size of these vesicles for both *B. divergens* and *B. microti* iRBCs spanned from 30 to 300 nm, although most vesicles fell in the 60–100 nm range. Furthermore, this size range has also been reported for other parasites, including Plasmodium (Mantel et al., 2013). A further complication in studying Babesia EVs is that host RBCs also secrete EVs, and thus, it is important to discriminate between the contribution from host and parasite. Looking at uRBC and iRBC profiles, we show that Babesia infection heightens EV production both *in vitro* and *in vivo* (**Figures 1A**,**2A**). The numbers of EVs have also been shown to increase during malaria infection both in patients and in experimental malaria models (Combes et al., 2004; Campos et al., 2010; Nantakomol et al., 2011; El-Assaad et al., 2014). Although a 10-fold increase has been reported for malaria infections, our data suggest a more modest elevation, approximating 4-fold in iRBCs over uRBCs (**Figure 1C**). However, both Babesia models argue for a direct correlation between parasitemia and concentration of EVs, with increasing parasitemias yielding higher concentrations of EVs, which could then yield the 10-fold increase seen in malaria. In fact, the plasma of *B. microti*–infected mice exhibited back to baseline numbers of vesicles (**Figure 2A**) as infection is cleared, in agreement with data from patients treated with antimalarials to clear the parasite (Nantakomol et al., 2011). Thus, quantification of EV numbers can aid screening of infection. Qualitatively, we also show that EVs are heterogeneous, in that a third of them can be identified by surface parasite markers. We further purified the EVs by density gradient ultracentrifugation and verified their identity by flow cytometry using classical markers (CD9 and CD81). The observation that >75% of particles in our purification are EVs, as also reported by other researchers working on EVs (Mellisho et al., 2017), confirms that our methodology of purification is efficient in enriching for EVs (**Supplementary Figure 1**). TEM confirmed this size property of the purified EVs. For EVs originating from *B. divergens* iRBCs, we show that Bd37 is present on a population of EVs whereas BMN-2 is present on a proportion of EVs purified from *in vivo B. microti*–infected plasma (**Figure 5**). Overall, our results argue for an augmentation of EV secretion with infection, and besides host proteins, parasite proteins are also present in and on these vesicles.

Although we have not fully characterized the parasite EV proteome, our immunoblot analysis of the contents from EVs from *B. microti*–infected plasma with immune sera from mice and humans showed that several EV parasite proteins were targets of the host humoral response (**Figure 4**). Such proteins can serve as the basis of both diagnostic and vaccine platforms. Previous efforts to identify proteins secreted by *B. microti* iRBCs focused on the screening of a *B. microti* cDNA library using sera from infected mice. These efforts resulted in the identification of only a limited number of small molecular weight proteins, the main contenders being members of the BMN family (Homer et al., 2000). Peptides from this family were used as the basis of an ELISA for *B. microti* as a screening test applied to endemic and non-endemic blood donor populations (Levin et al., 2014; Levin et al., 2016), with mixed results for sensitivity and specificity. Recent effort has been devoted to identifying the parasites in glycosylphosphatidylinositol-anchored proteome, and 19 proteins have been characterized as potential molecules that can be used to detect antibody response (Cornillot et al., 2016). The advantage of using EV-associated biomarkers is that these are extremely stable within the circulation, in the order of days (vs. minutes for traditional soluble markers), and that EVs are found in all biological fluids, making diagnosis less intrusive (Properzi et al., 2013).

Small RNA cargo is specifically enriched for a subset of the cell’s total small RNA pool (Nolte-’T Hoen et al., 2012). miRNAs are often associated with life cycle regulation, susceptibility to infection of host cells, and modulation of host innate immune responses (Buck et al., 2014; Retana Moreira et al., 2019). Thus, these non-coding RNA molecules are heavily involved in post-transcriptional gene regulation in most biological and pathological processes (Kim et al., 2015). We used a combination of microarray and NGS platforms to arrive at the different miRNA signatures obtained for EVs from uninfected and infected cultures (**Figures 6** and **7**). Both methods have their specific advantages and disadvantages (Willenbrock et al., 2009). Microarrays have been used extensively for the simultaneous profiling of thousands of genes in a single experiment. Along with quantitative real-time PCR, they are the most used platform to evaluate the expression of known miRNAs. They are relatively cost-effective, quick from RNA labeling to data generation, and simple to use. However, the short length of these molecules does not always allow for optimal probe design, as the miRNA sequences themselves must be used as the probe sequences. Based on these criteria, we also used the NGS platform in tandem to ensure a robust and more comprehensive expression profiling of miRNAs (Tam et al., 2014; Chatterjee et al., 2015). We first obtained miRNA profiles using microarrays because they can simultaneously profile thousands of sequences in a single experiment. However, only known miRNA species will be characterized. Sequencing allows the identification of novel miRNA species, and in fact, almost 200 novel miRNA species that had not been previously mapped to EVs were identified. More importantly, we found significant differences in both presence and concentration of specific miRNAs between EV contents from uRBCs and iRBCs. There were three highly upregulated miRNAs: miR-4534, miR-4463, and miR-7106-5p. Of these, the most significant difference was found to be with miR-4463, which was found to be present 20-fold times in uRBCs. Interestingly, miR-4463 is associated with apoptosis and oxidative stress in endothelial cells (Wang et al., 2017; He et al., 2018). Elevated oxidative stress in the host cells has been linked to intracellular parasite pathogens like M. tuberculosis and *P. falciparum* (Chawla et al., 2012; Beri et al., 2017; Beri et al., 2019), and our previous work had shown that *B. divergens*–infected red cells experience disturbed redox homeostasis. Thus, miRNAs may play a role in parasite-related oxidative stress, and future work will be directed toward understanding its role in potentially protecting the cell. Both our platforms detected an upregulation of miR-4454, which has been associated with severe thrombocytopenia in P. vivax–infected human plasma (Santos et al., 2021). Like malaria, babesiosis is also associated with a steep drop in platelets (Akel and Mobarakai, 2017; Ripoll et al., 2018). Furthermore, in our microarray platform, we also found an almost 3.5-fold increase in miR-4497 in iRBC-derived EVs. This miRNA has been associated with splenic sequestration in *P. falciparum* (Gupta et al., 2021; Gupta and Wassmer, 2021), but their role in Babesia infection is yet to be characterized. Overall, significant differences were found in the miRNA cargo of the EVs from uRBCs and iRBCs, and future experiments will help dissect out the role each of these play in the pathogenesis of disease. Furthermore, such differentially expressed EV-resident RNAs can serve as biomarkers, as these RNAs can be detected at extremely low quantities (Kim et al., 2017).

EV secretion by parasites has been linked to multiple functions including intercellular communication between host and parasite and between parasites (Coakley et al., 2015; Wu et al., 2018; Babatunde et al., 2020; Olajide and Cai, 2020; Torrecilhas et al., 2020; Opadokun and Rohrbach, 2021) as well as modulation of the host immune response (Sisquella et al., 2017; Kuipers et al., 2018; Dantas-Pereira et al., 2021; Drurey and Maizels, 2021). For EVs to serve as vehicles of communication between various types of cells, EVs need to be taken up by other cells. Using CFSE-labeled EVs and ImageStream analysis, we showed that EVs are taken up by both uRBCs and iRBCs, albeit at a slightly higher percentage in the latter (**Figures 3A–E**). As the time of co-incubation increases, the percentage of cells that take up EVs also increases. Therefore, circulating EVs in a culture system can be internalized by uRBCs and iRBCs, possibly leading to the transfer of biomolecules, which are known to be contained within EVs. Furthermore, we also showed that 90% of the monocytes, when co-incubated with purified *B. divergens* EVs, internalize these EVs (**Figure 3F**). As elaborated above, in other parasite systems, EVs are known to aid in immune evasion and manipulation of the microenvironment by the parasite, and future work is needed to examine this cellular crosstalk in Babesia.

Overall, our work provides important data for understanding the biological components of Babesia EVs and lays the foundation to future studies directed toward analyzing the consequences of EV cargo in determining the outcome of parasite infection.

## Data availability statement

The data presented in the study are deposited in NCBI repository with BioProject ID PRJNA874078 titled “miRNA analysis of EVs from Babesia divergens culture supernatant”.

## Ethics statement

The studies involving human participants were reviewed and approved by NYBC IRB. The patients/participants provided their written informed consent to participate in this study. All animal studies were approved by the New York Blood Center’s Animal Care and Use Committee.

## Author contributions

DB, MR, YL, KY, and CL conceived the experiments. DB, MS, MR, and GR purified the EVs from culture and plasma and performed uptake experiments. DB, YL, and MR performed the IFC experiments. DB, MR, XA, KY, and CL analyzed the results. DB, KY, and CL wrote the manuscript. All authors contributed to the article and approved the submitted version.

## Funding

This research was funded by National Institutes of Health Grants P01 HL149626 (KY and CL) and R01HL140625 (CL) and a grant from BNY Mellon (KY and CL).

## Acknowledgments

The authors acknowledge help from the Flow Cytometry Lab (RRID: SCR_021779) at Lindsley F. Kimball Research Institute, New York Blood Center.

## Conflict of interest

The authors declare that the research was conducted in the absence of any commercial or financial relationships that could be construed as a potential conflict of interest.

## Publisher’s note

All claims expressed in this article are solely those of the authors and do not necessarily represent those of their affiliated organizations, or those of the publisher, the editors and the reviewers. Any product that may be evaluated in this article, or claim that may be made by its manufacturer, is not guaranteed or endorsed by the publisher.
