# Supplementary material for: Identification and characterization of extracellular vesicles from red cells infected with Babesia divergens and Babesia microti
Source: Front Cell Infect Microbiol. 2022 Oct 7;12:962944. doi: 10.3389/fcimb.2022.962944 (PMC9585353; doi:10.3389/fcimb.2022.962944)
Supplement: Supplementary file 3 [file DataSheet_3.docx]

1. **Library construction and sequencing**

Total RNA was extracted using Trizol reagent (Invitrogen, CA, USA) following the manufacturer's procedure. The total RNA quality and quantity were analysis of Bioanalyzer 2100 (Agilent, CA, USA) with RIN number >7.0. Approximately 1 ug of total RNA were used to prepare small RNA library according to protocol of TruSeq Small RNA Sample Prep Kits (Illumina, San Diego, USA). And then we performed the single-end sequencing 50bp on an Illumina Hiseq 2500 at the LC Sciences (Hangzhou, China) following the vendor's recommended protocol.


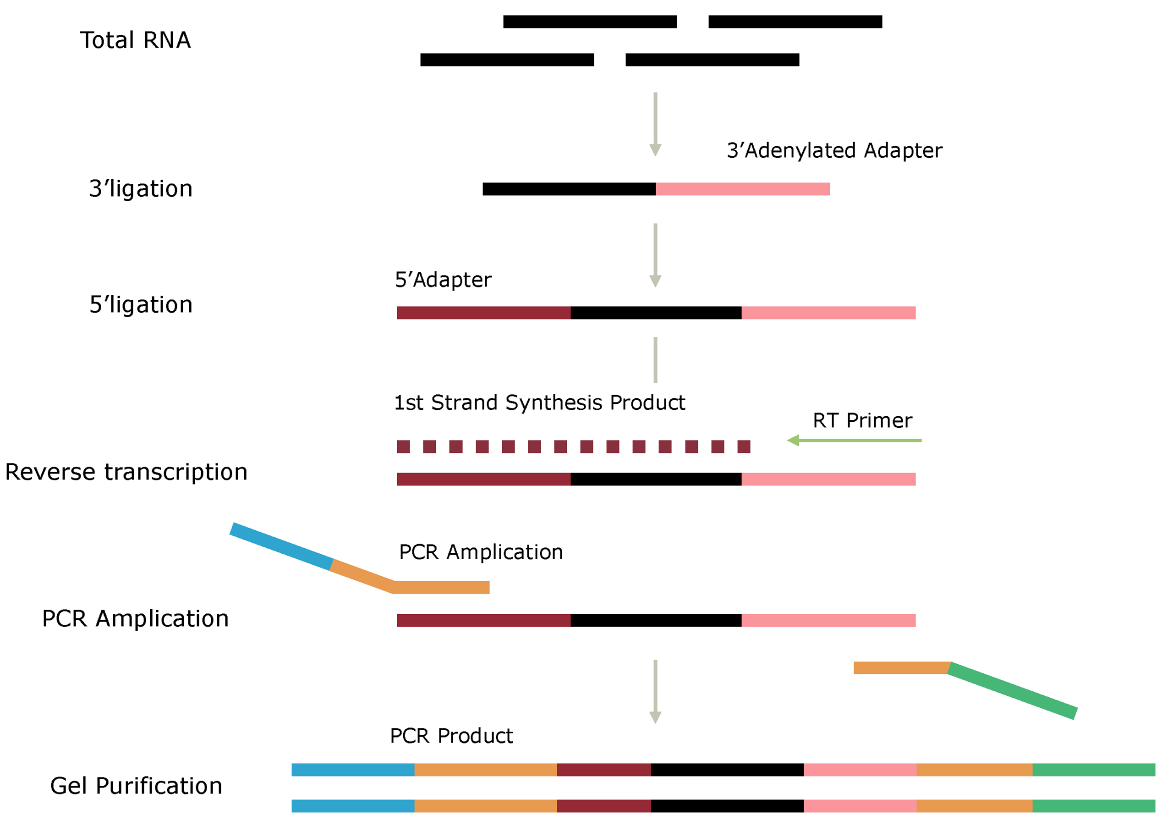


**Workflow of library construction**

**2. Bioinformatics analysis**

**Animal**: Raw reads were subjected to an in-house program, ACGT101-miR (LC Sciences, Houston, Texas, USA) to remove adapter dimers, junk, low complexity, common RNA families (rRNA, tRNA, snRNA, snoRNA) and repeats. Subsequently, unique sequences with length in 18~26 nucleotide were mapped to specific species precursors in miRBase 22.0 by BLAST search to identify known miRNAs and novel 3p- and 5p- derived miRNAs. Length variation at both 3’ and 5’ ends and one mismatch inside of the sequence were allowed in the alignment. The unique sequences mapping to specific species mature miRNAs in hairpin arms were identified as known miRNAs. The unique sequences mapping to the other arm of known specific species precursor hairpin opposite to the annotated mature miRNA-containing arm were considered to be novel 5p- or 3p derived miRNA candidates. The remaining sequences were mapped to other selected species precursors (with the exclusion of specific species) in miRBase 22.0 by BLAST search, and the mapped pre-miRNAs were further BLASTed against the specific species genomes to determine their genomic locations. The above two we defined as known miRNAs. The unmapped sequences were BLASTed against the specific genomes, and the hairpin RNA structures containing sequences were predicated from the flank 80 nt sequences using RNAfold software (<http://rna.tbi.univie.ac.at/cgi-bin/RNAWebSuite/RNAfold.cgi>). The criteria for secondary structure prediction were: (1) number of nucleotides in one bulge in stem (≤12) (2) number of base pairs in the stem region of the predicted hairpin (≥16) (3) cutoff of free energy (kCal/mol ≤-15) (4) length of hairpin (up and down stems + terminal loop ≥50) (5) length of hairpin loop (≤20). (6) number of nucleotides in one bulge in mature region (≤8) (7) number of biased errors in one bulge in mature region (≤4) (8) number of biased bulges in mature region (≤2) (9) number of errors in mature region (≤7) (10) number of base pairs in the mature region of the predicted hairpin (≥12) (11) percent of mature in stem (≥80).

**Plant**: Raw reads were subjected to an in-house program, ACGT101-miR (LC Sciences, Houston, Texas, USA) to remove adapter dimers, junk, low complexity, common RNA families (rRNA, tRNA, snRNA, snoRNA) and repeats. Subsequently, unique sequences with length in 18~25 nucleotide were mapped to specific species precursors in miRBase 22.0 by BLAST search to identify known miRNAs and novel 3p- and 5p- derived miRNAs. Length variation at both 3’ and 5’ ends and one mismatch inside of the sequence were allowed in the alignment. The unique sequences mapping to specific species mature miRNAs in hairpin arms were identified as known miRNAs. The unique sequences mapping to the other arm of known specific species precursor hairpin opposite to the annotated mature miRNA-containing arm were considered to be novel 5p- or 3p derived miRNA candidates. The remaining sequences were mapped to other selected species precursors (with the exclusion of specific species) in miRBase 22.0 by BLAST search, and the mapped pre-miRNAs were further BLASTed against the specific species genomes to determine their genomic locations. The above two we defined as known miRNAs. The unmapped sequences were BLASTed against the specific genomes, and the hairpin RNA structures containing sequences were predicated from the flank 120 nt sequences using RNAfold software (<http://rna.tbi.univie.ac.at/cgi-bin/RNAWebSuite/RNAfold.cgi>). The criteria for secondary structure prediction were: (1) number of nucleotides in one bulge in stem (≤12) (2) number of base pairs in the stem region of the predicted hairpin (≥16) (3) cutoff of free energy (kCal/mol ≤-15) (4) length of hairpin (up and down stems + terminal loop ≥50) (5) length of hairpin loop (≤200). (6) number of nucleotides in one bulge in mature region (≤4) (7) number of biased errors in one bulge in mature region (≤2) (8) number of biased bulges in mature region (≤2) (9) number of errors in mature region (≤4) (10) number of base pairs in the mature region of the predicted hairpin (≥12) (11) percent of mature in stem (≥80).

**Bioinformatics pipeline for miRNA**


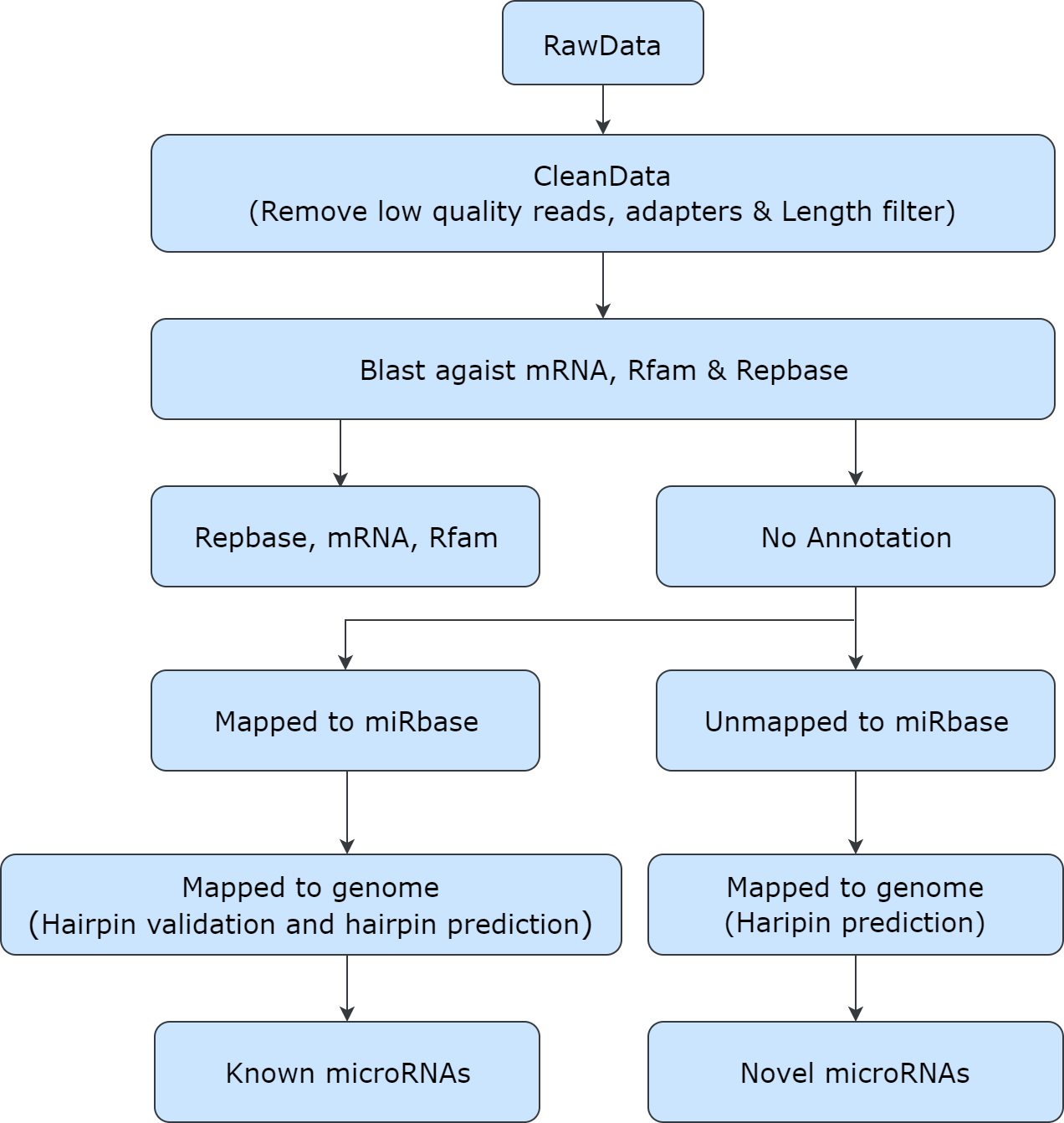


**3. Database**

| **Dababase** | **Web links** | **Version/date** |
| --- | --- | --- |
| species priority | [hsa](file:///J:\Bloodborne%20Parasites\DivyaEVpaper\Microarray%20mirna\MV-Sequencing%20microRNA%20Extract\Report\8202_RM_report\hsa) |  |
| miRNA/Pre-miRNA database (miRbase) | [ftp://miRbase.org/pub/miRbase/CURRENT](ftp://miRbase.org/pub/miRbase/CURRENT/) | 22.0 |
| Rfam | <http://rfam.janelia.org> | 13.0 |
| Repbase | <http://www.girinst.org/repbase> | 2017.09 |
| Genome | <ftp://ftp.ensembl.org/pub/release-101/fasta/homo_sapiens/dna/> |  |
| Gene Ontology (GO) | <ftp://ftp.ncbi.nih.gov/gene/DATA/gene2go.gz> | 2016.04 |
| KEGG | <http://www.genome.jp/kegg/pathway.html> | 2017.06 |
| eggNOG | <http://eggnogdb.embl.de/> | 4.5 |
|  |  |  |

**4. Bioinformatics software**

Small RNA sequencing data were analyzed by in-house program ACGT101-miR.
