# Supplementary material for: Identification and characterization of extracellular vesicles from red cells infected with Babesia divergens and Babesia microti
Source: Front Cell Infect Microbiol. 2022 Oct 7;12:962944. doi: 10.3389/fcimb.2022.962944 (PMC9585353; doi:10.3389/fcimb.2022.962944)
Supplement: Supplementary file 4 [file DataSheet_4.docx]

**Supplementary File – 4**

**Identification and Characterization of Extracellular Vesicles from Red Cells Infected with *Babesia divergens* and *Babesia microti***

Divya Beri ^1^, Marilis Rodriguez ^1^, Manpreet Singh ^1^, Yunfeng Liu ^2^, Giselle Rasquinha ^3^, Xiuli An ^4^, Karina Yazdanbakhsh ^2^ and Cheryl A. Lobo ^1*^

^1^ Department of Blood-Borne Parasites

^2^ Department of Complement Biology

^4^Department of Membrane Biology,

Lindsley F. Kimball Research Institute, New York Blood Center, New York, New York,

^3^Georgetown University, 3700 O St NW, Washington, DC

***Corresponding author:**

[CLobo@Nybc.org](mailto:CLobo@Nybc.org)

Tel: 212-570-3415

**Flow cytometry-based confirmation of identity of EVs purified by ultracentrifugation**

Flow cytometry was used to verify the purity of EVs post ultracentrifugation using classical markers CD9 and CD81. The following reagents were used for the assay.

**Capture antibodies**

1. CD9 (https://www.thermofisher.com/order/catalog/product/10620D)
2. CD81(https://www.thermofisher.com/order/catalog/product/10622D)

**Detection antibodies linked to PE fluorophore**

1. CD9 (https://www.citeab.com/antibodies/2413810-555372-bd-pharmingen-pe-mouse-anti-human-cd9)
2. CD81 (https://www.citeab.com/antibodies/2412825-555676-bd-pharmingen-pe-mouse-anti-human-cd81)


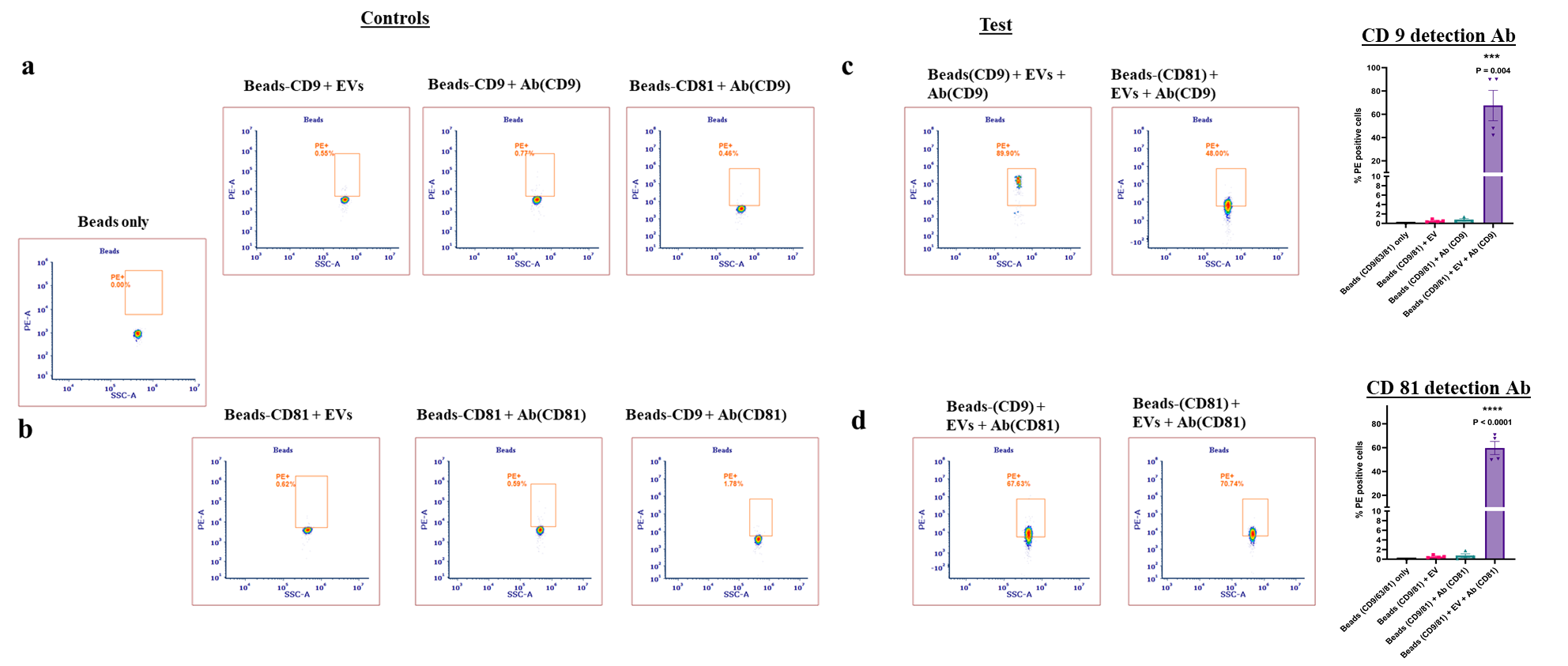


**Supplementary Fig. 1: Flow cytometry-based confirmation of identity of EVs purified by ultracentrifugation using classical markers.** As described under Methods, magnetic beads coated with CD9 or CD81 (capture antibodies) were used and incubated with EVs followed by staining with CD9 or CD81 detection antibodies conjugated with fluorophore PE. (a) and (b) show the controls used in the experiment and were used for appropriate gating. (c) shows the PE positivity using CD9 detection antibody and shows > 70% events were PE+ (n = 2 for each capture antibody). (d) shows the PE positivity using CD81 detection antibody and shows > 60% events were PE+ (n = 2 for each capture antibody).

**Gating strategy for experiments involving internalization of CFSE-labelled EVs in RBCs**

The following was the gating strategy used in IDEAS™ software provided by Amnis Imagestream. (Please also refer to Methods, Results section 2 and Fig. 3).


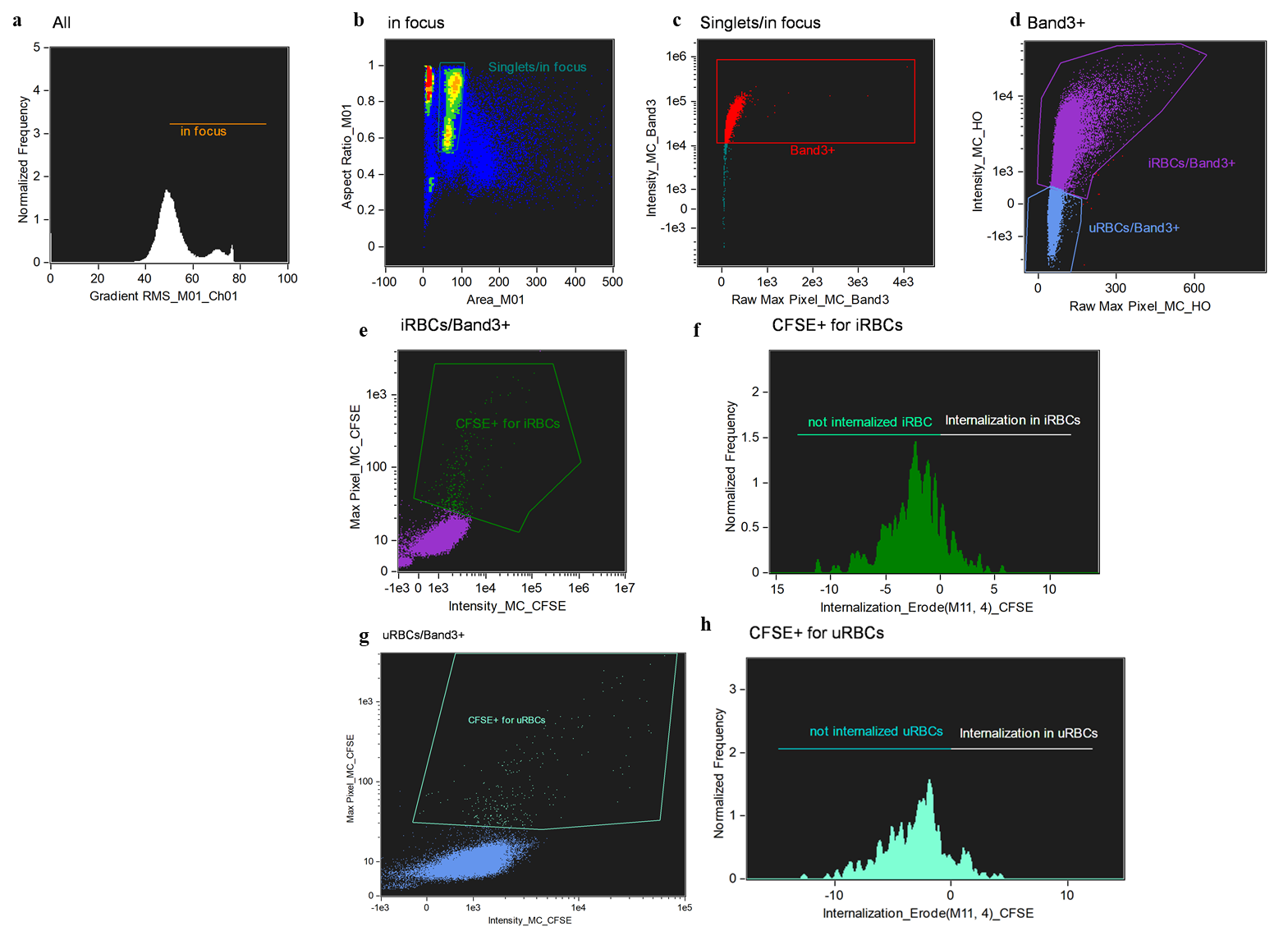


**Supplementary Fig. 2: Gating strategy used for demonstrating internalization of CFSE-labelled EVs in RBCs infected with B. divergens.** (a) Using Gradient RMS of Ch01 (BF), cells in focus ( > 50 on X-Axis) were gated. (b) In focus cells were further gated for Singlets. All doublets and cells with aspect ration < 0.5 were excluded from analysis. (c) Singlets were further gated for the red cell marker Band3-APC. (d) Band3 positive cells were separated on basis of Hoechst (HO) signal into HO+ (gated iRBCs) and HO- (gated uRBCs). (e) CFSE+ cells were gated in iRBCs parent gate. (f) Internalization wizard of IDEAS™ software was used to identify Band3+, HO+ (iRBCs) with internalized CFSE labelled EVs. (g) CFSE+ cells were gated in uRBCs parent gate. (f) Internalization wizard of IDEAS™ software was used to identify Band3+, HO- (uRBCs) with internalized CFSE labelled EVs.
